# The effect of aging on the repeated-dose liver micronucleus assay

**DOI:** 10.1186/s41021-021-00212-3

**Published:** 2021-09-09

**Authors:** Miyuki Shigano, Hironao Takasawa, Shuichi Hamada

**Affiliations:** 1 Safety Assessment Department, Kashima Laboratories, LSIM Safety Institute Corporation, 14-1 Sunayama, Kamisu-shi, Ibaraki, 314-0255 Japan; 2 Bozo Research Center Inc, 1-3-11, Hanegi, Setagaya-ku, Tokyo, 156-0042 Japan

**Keywords:** Liver micronucleus assay, Collagenase, Hepatocyte, Repeated-dose liver micronucleus assay, Clofibrate

## Abstract

**Background:**

The liver micronucleus (MN) assay is an effective and important in vivo test for detecting genotoxic compounds. In particular, the repeated-dose liver MN (RDLMN) assay which greatly facilitates incorporation of the liver MN assay into the general toxicity study has been developed. Usefulness of the RDLMN assay was appraised highly in the 7th International Workshops on Genotoxicity Testing (2017 in Tokyo) in that sufficient numbers and types of chemicals were studied and easy integration into the general toxicity study is preferred from the 3R’s point of view. However, it was pointed out that it is necessary to evaluate the effect of age at the start of 4-week repeated administration, since there are limited data, where only those of rats of 6 week of age at the start of administration are available.

In this study, we conducted the 4-week RDLMN assay using rats of 6 and 8 weeks of age (at the start of administration) to investigate the effect of age on the liver MN inducibility. Clofibrate, a weak inducer of liver MN, was used in this study to detect the slight difference in the liver MN induction.

**Results:**

The liver MN induced by clofibrate was detected in both rats of 6 and 8 weeks of age at the start of administration. However, the liver MN induction was lower in rats of 8 weeks of age compared to rats of 6 weeks of age at the start of administration.

**Conclusion:**

These results suggest that the liver MN inducibility decreases with age. Therefore, we recommend the use of rats of 6 weeks of age at start of administration to reliably detect the liver MN induction in the RDLMN assay.

## Introduction

The liver MN assay is useful to evaluate the genotoxic potential to induce chromosome aberration in the liver. Especially, the repeated-dose liver MN (RDLMN) assay has been developed well in the Mammalian Mutagenicity Study Group (MMS) in the Japanese Environmental Mutagen Society (JEMS) because it could be incorporated into the general toxicity study. In the Collaborative Study Group for the Micronucleus Test (CSGMT) of JEMS/MMS, 22 carcinogens were evaluated in the RDLMN assay, in which hepatocarcinogens showed a positive response in the liver [1, 2]. In additional tests, aneugens showed a positive response in the liver and non-carcinogens showed a negative response in the RDLMN assay. These results were reported in the 7th International Workshop on Genotoxicity Testing (IWGT, 2017 in Tokyo), and the working group agreed that the RDLMN assay is sufficiently validated for an OECD guideline in terms of numbers and types of chemicals studied [3]. However, some issues were pointed out; it is necessary to evaluate the effect of age (6 weeks versus older) at the start of administration. This is because some countries, especially Western countries, conduct the general toxicology study using rats older than 6 weeks at start of administration, whereas the CSGMT described above used only rats of 6 weeks of age at the start of administration.

Clofibrate is an exogenous ligand of peroxisome proliferator-activated receptor alpha (PPARα), and it is suggested that clofibrate generates reactive oxygen species through PPARα and induces liver cancer in rats. Clofibrate showed a weak liver MN induction in the 2-week and 4-week RDLMN assays using rats of 6 weeks of age at the start of administration [4].

We conducted the 4-week RDLMN assay using rats of 6 and 8 weeks of age at the start of administration to examine the effect of age on the results of the RDLMN assay. Clofibrate, a weak inducer of the liver MN, was used in this study to detect the slight difference in the liver MN induction.

## Materials and methods

### Animals

Male Crl:CD (SD) rats aged 5 and 7 weeks were purchased from Charles River Japan Inc. (Yokohama, Japan) and were aged 6 and 8 weeks at the start of administration. The animals were housed two to three per cage in an air-conditioned room with a 12-h light/dark cycle and free access to food and drinking water. The experimental protocol was approved by the Institutional Animal Care and Use Committee prior to its implementation.

### Chemicals

Clofibrate (CAS No. 637–07-0, > 98.0% purity) was purchased from Wako Pure Chemical Industries, Ltd. (Tokyo, Japan). Clofibrate was suspended in corn oil (Wako Pure Chemical Industries, Ltd.). The vehicle was used as the control substance.

### Dose levels and treatment schedules

Clofibrate was administered orally to rats (*n* = 5/group) at 0 (vehicle alone), 125 mg/kg, and 500 mg/kg for 28 consecutive days. All dose volumes were set at 10 mL/kg/day. All animals were checked daily for clinical signs and weighed on Days 1, 8, 15, 22, and 29. The date of the first administration of the test chemical was regarded as Day 1.

### Liver micronucleus assay

Twenty-four hours after the last administration, rats (10 and 12 weeks of age) were euthanized under thiopental anesthesia. Then, the liver were removed from each rat and the central part of lateral left lobe was used for the liver MN assay as previously reported [1, 2, 5]. Briefly, a small portion of the liver tissue (approximately 1 to 2 g around the central part of the lateral left lobe) was sliced at about 2 mm with a razor blade and washed well with physiological saline. The sliced liver was incubated in about 20 mL of a collagenase solution (containing Collagenase Yakult: 40–50 U/mL Yakult Pharmaceutical Industry Co., Ltd., Tokyo, Japan, Lot No.: 121026–01) at 37 °C for 1 h while being shaken at approximately 50 rpm. After the incubation, the liver tissues and the collagenase solution were mixed vigorously to isolate the hepatocytes (HEPs). The mixture was filtered through gauze and a cell strainer (pore size: 100 μm). The HEP suspension was centrifuged at 50×*g* for 2 min and the supernatant was discarded. About 20 mL of 10% phosphate buffered formalin was added to the pellet. The centrifugation was repeated and the supernatant was discarded. The pellet of the HEPs was mixed with 10% phosphate-buffered formalin to prepare an HEP-suspension. Just before microscopic observation, the prepared HEP-suspension was mixed and stained with the same volume of a staining solution containing acridine orange (AO) at 500 μg/mL and 4′,6-diamidino-2-phenylindole dihydrochloride (DAPI) at 10 μg/mL (AO-DAPI). The mixtures were dropped onto clean glass slides and spread with coverslips. The slide specimens were observed under a fluorescent microscope (BX51N-34-FL-1-D; Olympus Corporation, Tokyo, Japan) with a UV-excitation filter (wave length: 330–385 nm) and an emission filter (wave length: 420 nm).

### Calculation of micronucleus incidences and mitotic indices, and statistics

The incidence of micronucleated HEPs (MNHEPs) was calculated by counting 2000 HEPs for each animal. In addition, the number of mitotic phase cells in 2000 HEPs was counted to determine the mitotic index (MI). Differences in the incidence of MNHEPs between the clofibrate-treated and vehicle control groups were analyzed using the conditional binomial test of Kastenbaum and Bowman [6] for each age group. To evaluate the difference in sensitivity, the concentrations of clofibrate and MNHEP (%) were fit to the regression line using the least squares regression (Microsoft Excel 2016, Microsoft Corporation). Body weights were using Bartlett’s test for homogeneity of variance among groups. Since the variances were homogeneous in both the ages of treated animals, Dunnett’s parametric test was applied. Significance was evaluated at the 5% or 1% level using a two-tailed test for increases or decreases relative to the vehicle control group for each age group.

## Results

### Clinical sign and body weights

**Fig. 1:**
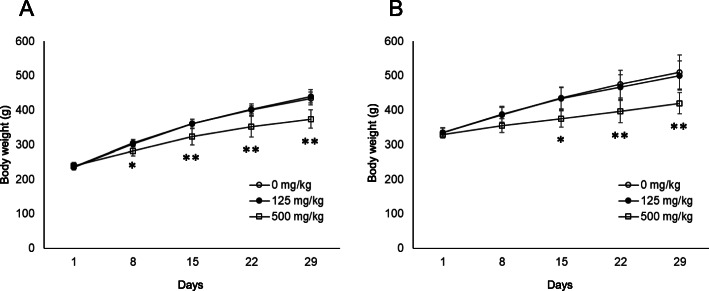
Comparison of body weights of rats of 6 and 8 weeks of age at the start of administration using clofibrate. Body weights (g); Comparison between the vehicle group and clofibrate-treated group in rats of 6 weeks of age (**A**) and rats of 8 weeks of age (**B**) at the start of administration. Values are presented as the mean and SD. Differences in the body weights between the test and vehicle control groups were analyzed by the Dunnett test at significance levels of 5 and 1% (*: *P* < 0.05, **: *P* < 0.01)

In rats of 6 weeks of age at the start of administration, the body weights in the 500 mg/kg group were significantly lower than that in the control group on and after Day 8 (Fig. 1A). In the 500 mg/kg group, staggering gait and salivation were observed from Days 1 to 7 and from Days 13 to 28, respectively (data not shown). On the other hand, in rats of 8 weeks of age at the start of administration, the body weights in the 500 mg/kg group were significantly lower than that in the control group on and after Day 15 (Fig. 1B). In the 500 mg/kg group, staggering gait and salivation were observed from Days 1 to 4 and Days 15 to 28, respectively (data not shown).

### Comparison of incidences of micronucleated hepatocytes and indices of M-phase in hepatocytes

**Fig. 2:**
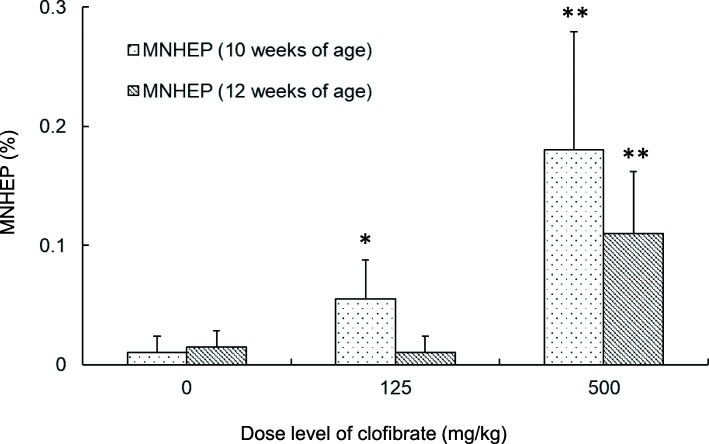
Comparison of RDLMN assay results of rats of 10 and 12 weeks of age at the end of administration using clofibrate. Incidences of MNHEPs (%); Comparison between the vehicle group and clofibrate-treated group in rats of 10 and 12 weeks of age at the end of administration. Differences in the incidences of MNHEPs between the test and vehicle control groups were analyzed by the Kastenbaum and Bowman test at significance levels of 5 and 1% (*: *P* < 0.05, **: *P* < 0.01)

**Fig. 3:**
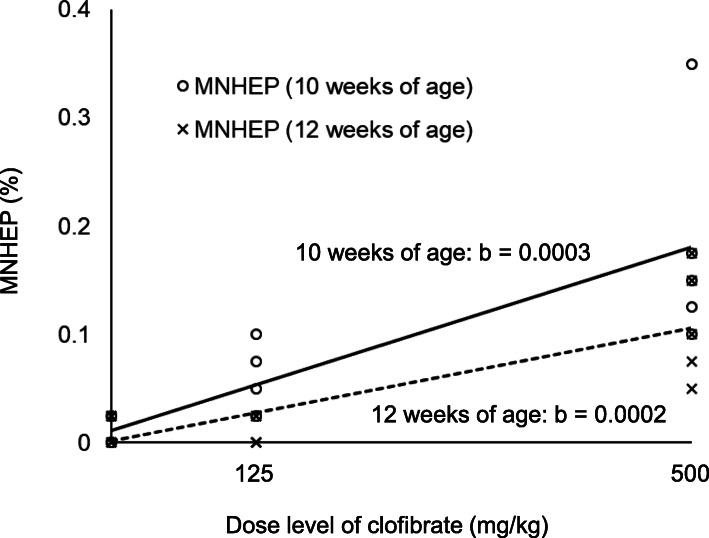
Comparison of sensitivity of RDLMN assay of rats of 10 and 12 weeks of age at the end of administration using clofibrate. Individual values of the incidence of MNHEPs (%) and the regression line (solid line: rats of 10 weeks of age at the end of administration, dotted line: rats of 12 weeks of age at the end of administration). The b values are estimates of the slope

In rats of 10 weeks of age at the end of administration (6 weeks of age at the start of administration), the incidences of MNHEPs in the 125 and 500 mg/kg groups were significantly higher than that in the control group (Fig. 2). The indices of M phase in hepatocytes (M phase, %) were 0.000 ± 0.000%, 0.005 ± 0.011%, and 0.010 ± 0.022% at 0, 125 and 500 mg/kg, respectively. On the other hand, in rats of 12 weeks of age at end of administration (8 weeks of age at the start of administration), the incidences of MNHEPs were significantly higher only in the 500 mg/kg group than that in the control group (Fig. 2). There were no M phase hepatocytes (M phase: 0.000 ± 0.000% in all groups). The sensitivity to clofibrate in rats of 12 weeks of age at the end of administration (slope: 0.0002) was lower than that in rats of 10 weeks of age at the end of administration (slope: 0.0003, Fig. 3).

## Discussion

At the 7th IWGT, it was pointed out that it would be necessary to evaluate the effect of age (6 weeks versus older) since most of the liver MN validation studies used rats of 6 weeks of age at the start of administration whereas rats of 8 weeks of age are used for the general toxicology study in many laboratories [3]. Micronuclei formation requires cell proliferation. The effect of age on the micronucleus assay has also been reported in the bone marrow MN assay, and are thought to be due to changes in cell proliferation ability with aging [7]. In the liver MN assay, MN induction has been evaluated using juvenile rats (4 weeks old) with active hepatocyte proliferation [8–11].

The repeated dose method enables MN induction in adult rats by utilizing accumulation of hepatocytes with micronuclei, but the effect of age needs to be examined. In this study, a 4-week RDLMN assay was conducted to evaluate the effect of age on the liver MN induction using rats of 6 and 8 weeks of age at the start of administration. To detect slight difference in liver MN induction, we selected clofibrate, a weak liver MN inducibility and dosed it to rats. As a result, liver MN induction was detected in both rats of 6 and 8 weeks of age at the start of administration. Although there were individual differences in MNHEP (%) in rats of 6 weeks of age at the start of administration, it is considered that there are some differences in sensitivity due to age. The differences due to age in the liver MN induction are likely caused by mitotic activity. However, M phase hepatocytes were rarely observed at specimen preparation (4 weeks after the start of administration) in rats of 10 and 12 weeks of age at the end of administration. It suggests that the differences in mitotic activity were due to the age of animals at the start of administration and cell division decreases with age. This implies that younger animals are more sensitive to liver MN induction as they showed a higher rate of hepatocyte proliferation, although the rates of replicative DNA synthesis were similar in rats aged from 5 to 8 weeks as previously reported [12]. Since MNHEPs were accumulated in the liver during 28-day administration, these can be detected if a chemical has some liver MN inducibility like clofibrate, but these may not be detected if a chemical has less inducibility in animals with less mitotic activity; i.e. rats of 8 weeks of age at the start of administration. Also, since clofibrate is metabolized by CYP, there are likely effects of age on CYP. Asaoka et al. [13] reported that the total CYP content was low in rats of postnatal day 4, and increased with age. Although the toxicity assessment parameters other than clinical sign observation and body weight measurement were not evaluated in this study, it is possible that differences in CYP expression or activity caused differences in toxicity, which lead to differences in MNHEP induction.

In conclusion, both rats of 6 and 8 weeks of age at the start of administration can be used for the RDMN assay, however, the sensitivity is considered to be higher in rats of 6 weeks of age. Further studies with several compounds may be necessary to determine usability of rats of 8 weeks of age at the start of administration in the RDLMN assay.

## Data Availability

All date generated or analyzed during this study are included in this published article.

## Abbreviations

RDLMN assay: Repeated-dose liver micronucleus assay
CSGMT: The Collaborative Study Group for the Micronucleus Test
JEMS: The Japanese Environmental Mutagen Society
MMS: Mammalian Mutagenicity Study Group
MNHEP: Micronucleated hepatocyte
HEP: Hepatocyte
